# Study on the Self-Healing Performance of Polyurethane/Graphene Oxide-Modified Asphalt Based on Dynamic Disulfide Bonds

**DOI:** 10.3390/ma18112549

**Published:** 2025-05-29

**Authors:** Guokai Li, Min Wang, Kezhen Yan, Xiaojin Song

**Affiliations:** 1 College of Civil Engineering, Hunan University, Changsha 410082, China; lgk@hnu.edu.cn (G.L.); wangmin19@hnu.edu.cn (M.W.); 2 Key Laboratory for Green and Advanced Civil Engineering Materials and Application Technology of Hunan Province, Changsha 410082, China; 3 Zhongteng Zhixin Technology Company of Hunan Province, Changsha 410082, China; sxjmail@126.com

**Keywords:** polyurethane, graphene oxide, disulfide bonds, self-healing asphalt

## Abstract

In this study, an investigation on using polyurethane/graphene oxide (PU/GO) containing disulfide bonds as a modifier to improve the self-healing ability of asphalt was conducted. PU/GO with different GO contents were synthesized and modified asphalt with different PU/GO dosages (2%, 4%, 6%, 8%) were also prepared. The effect of GO contents on the mechanical and self-healing properties of PU/GO was explored and the impacts of PU/GO contents on the basic properties and self-healing properties of asphalt were also investigated. The results indicated that GO could significantly improve the mechanical properties of PU, as the tensile strength of PU/GO with 1.6% GO increased by more than 100% compared with pure PU. Moreover, GO also had a positive impact on the early-stage self-healing properties of PU/GO. PU/GO could be well dispersed in asphalt and clearly improve the low-temperature performance of base asphalt. When the PU/GO content is 8%, the ductility of modified asphalt was almost 6 times that of base asphalt. The results of both ductility and BBR self-healing tests revealed that the addition of PU/GO improved the self-healing properties of asphalt under room temperature and infrared light conditions. Especially under infrared light conditions, the ductility self-healing coefficient of 8% PU/GO-modified asphalt could reach 100% after healing for 15 min.

## 1. Introduction

Asphalt pavement has gradually become the main choice for high-grade pavement in recent years due to its advantages such as low noise, high pavement smoothness, low maintenance cost and fast construction [1,2,3]. However, during the service of asphalt pavement, asphalt pavement may suffer from potholes, rutting, cracks and other faults due to the influence of the environment and traffic loads [4,5]. Because of the formation of cracks, rainwater and dust can seep into the interior of the pavement structure. From this, more serious faults will occur, which will greatly reduce the service life of the asphalt pavement [6]. Therefore, it is necessary to carry out continuous maintenance to extend the service life of asphalt pavement, which will consume a lot of energy and economy [7,8]. To reduce energy consumption and economic expenditure, scholars have begun to focus on self-healing asphalt and asphalt mixtures. Although asphalt has a certain self-healing ability under natural conditions, it is not sufficient to repair cracks caused by traffic loads or environmental changes in a timely manner. Therefore, it is necessary to improve the self-healing ability of asphalt to prolong the service life of asphalt pavement [9,10].

At present, the most common and widely used methods to increase the self-healing property of asphalt include rejuvenator encapsulation, induction heating and microwave heating methods [11,12,13]. The rejuvenator encapsulation method needs to encapsulate the rejuvenator into a microcapsule and then mix the microcapsule with an asphalt mixture. The microcapsules will rupture when a crack appears, and the rejuvenator will be released to fill and repair the crack [14,15,16]. The induction heating method needs to add metal fibers or other conductive materials to asphalt, and then use electromagnetic induction to generate energy inside the asphalt, which will heat the asphalt material and heal the cracks. Through induction heating, the internal temperature of the asphalt mixture can be effectively increased, which will increase the self-healing performance of the asphalt mixture [17,18]. What is more, indoor tests have shown that using induction heating technology on asphalt mixtures can increase the service life of asphalt pavements by 30% [19]. However, it is worth noting that induction heating can only be applied to cracks within a certain width range [20]. Compared with induction heating, microwave heating has better heating efficiency and a wider range of applications [21,22]. Moreover, the microwave heating effect is more significant and the self-healing property of asphalt will be better when a smaller amount of metal materials are added to asphalt. [23,24].

Although the above methods can effectively enhance the self-healing ability of asphalt mixtures, there are still shortcomings in practical applications. Microcapsule technology can effectively improve the self-healing ability of asphalt pavement cracks, but the rejuvenator in microcapsules is limited. When cracks occur again in the same location, the self-healing property of asphalt will decrease due to the decrease in microcapsules. Induction heating and microwave heating methods can repair cracks multiple times. However, many microcracks are difficult to detect promptly, so it is easy to miss the best repair opportunity. Based on the shortcomings of current methods, scholars have begun to improve the self-healing property by introducing dynamic covalent bonds into asphalt.

Li et al. [25,26] added PU-containing dynamic disulfide bonds to asphalt for modification. The results revealed that the self-healing PU had good compatibility with asphalt. The presence of disulfide bonds provided a foundation for improving the self-healing capability. Researchers also [27,28] evaluated the crack healing ability of PU-modified asphalt including dynamic disulfide bonds. The results showed that the presence of dynamic disulfide bonds could effectively increase the self-healing ability of microcracks in asphalt. Zhang [29] synthesized S-SBS materials with dynamic covalent bonds by introducing disulfide bonds into the SBS structure, and then modified asphalt with S-SBS. The results indicated that the crack resistance and fracture healing ability were improved by introducing disulfide bonds into asphalt. In addition to disulfide bonds, diselenide bonds also have excellent dynamic exchangeability. Lyu et al. [30] synthesized a kind of PU with dynamic diselenide bonds and applied it to asphalt modification. The results showed that dynamic diselenide bonds endowed asphalt with excellent fracture self-healing abilities. Yang et al. [31] prepared modified asphalt containing dynamic disulfide and diselenide bonds, respectively, and compared the self-healing properties of these two types of modified asphalt. The results revealed that diselenide bonds had a greater effect on the short-term healing ability of asphalt, while the disulfide bonds had a greater effect on the long-term healing ability.

Based on the above research, it has been found that introducing dynamic covalent bonds into asphalt to enhance its self-healing ability is a feasible method. However, the recombination of dynamic covalent bonds is affected by temperature, and the higher the temperature, the higher the recombination efficiency of dynamic covalent bonds [27,28,29,30,31]. Researchers have found that graphene has excellent thermal conductivity and infrared absorption ability. Therefore, introducing graphene into PU can improve the heat absorption efficiency of PU. Higher temperatures can enhance the efficiency of covalent bond recombination and improve the self-healing ability of PU [32,33]. Xu et al. [34] even used graphene to modify asphalt mixtures. The results showed that graphene had excellent heat absorption ability, which could increase the surface temperature of asphalt mixtures and enhance the self-healing performance. Compared with graphene, graphene oxide (GO) also has excellent thermal conductivity and infrared absorption ability. At the same time, GO contains a large number of active functional groups, which can react with isocyanate in PU and have better dispersibility in PU. Li et al. [35] designed and synthesized self-healing PU/GO materials with different GO contents through the Diels Alder (D-A) reaction. The results showed that the material had excellent self-healing function under heating, infrared, and microwave conditions.

Based on the above discussion, it can be concluded that introducing dynamic covalent bonds into asphalt to improve the self-healing ability is a feasible method. Graphene or GO has excellent thermal conductivity and infrared absorption ability, which can improve the heat absorption efficiency and enhance the self-healing function. However, there is still a lack of research on the synergistic improvement of asphalt self-healing performance by dynamic covalent bonds and GO. Therefore, this study synthesized a kind of thermoplastic polyurethane containing dynamic covalent bonds, and then introduced GO into the PU to synthesize PU/GO composite materials. To explore the influence of GO on the self-healing function of PU under different conditions, two kinds of self-healing conditions were selected: heating condition at 60 °C and infrared light condition. Moreover, the effect of healing time on the self-healing property of PU/GO was also studied. Finally, PU/GO was added to asphalt to prepare modified asphalt with different PU/GO contents. In order to evaluate the self-healing performance of modified asphalt, self-healing tests based on ductility and BBR tests were conducted. Two kinds of self-healing conditions (25 °C room temperature and infrared light condition) were chosen to explore the effect of PU/GO on the self-healing property of asphalt under different conditions. In this study, the utilization of GO to improve the recombination efficiency of dynamic covalent bonds in PU and PU/GO-modified asphalt was investigated, which could provide a reference for the development of self-healing PU and asphalt materials.

## 2. Material and Test Methods

### 2.1. Raw Material

#### 2.1.1. Polytetramethylene Ether Glycol (PTMEG)

PTMEG is the most commonly used polyether diol in PU synthesis. The physical properties of PTMEG will vary with different molecular weights. In this study, PTMEG2000 produced by Macklin Chemical Reagent Co., Ltd. (Shanghai, China) was chosen for PU synthesis. The properties of PTMEG are shown in Table 1.

#### 2.1.2. Isophorone Diisocyanate (IPDI)

IPDI is a type of aliphatic diisocyanate with excellent light resistance and low toxicity. The molecular chains of PU synthesized by IPDI have stronger mobility, which is beneficial for the recombination of dynamic covalent bonds. Therefore, IPDI produced by Macklin Chemical Reagent Co., Ltd. was chosen for PU synthesis in this study and the properties of IPDI are shown in Table 2.

#### 2.1.3. 3,3′-Dithiodipropionic Acid (DPA)

To prepare self-healing PU, it is necessary to introduce dynamic covalent bonds into polyurethane. DPA is a type of aliphatic chain extender containing dynamic disulfide bonds. Therefore, DPA produced by Macklin Chemical Reagent Co., Ltd was selectedand the basic properties are given in Table 3. 

#### 2.1.4. Graphene Oxide (GO)

The main methods for preparing GO include the Brodie method, Staudenmaier method, and Hummers method [36]. The GO used in this study was prepared by the Hummers method.

#### 2.1.5. Base Asphalt

The base asphalt used for preparing PU/GO-modified asphalt is 70 pen asphalt (70#). The basic indices of 70# asphalt were tested according to the specification JTG E20-2011 [37]. The indices of the base asphalt are given in Table 4 and all the indices meet the requirements of the specification.

### 2.2. Sample Preparation

#### 2.2.1. The Preparation of PU and PU/GO

The synthesis methods of PU are divided into the one-step method and the pre-polymer method. Compared with the one-step method, the reaction process of the pre-polymer method is easier to control. There are fewer side reactions during the reaction process, so the final chemical structure of the molecular chains can be controlled. Therefore, the pre-polymer method was chosen to synthesize PU and PU/GO and the recipes of PU and PU/GO are given in Table 5.

**Figure 1:**
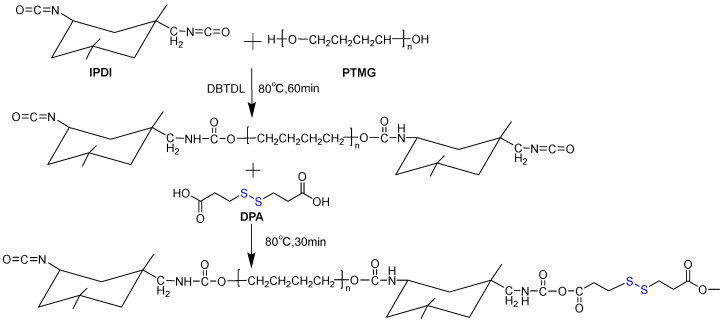
The reaction equation of PU.

Synthesis for PU: Considering the self-healing ability of PU, a hard segment content of 20% was selected in this study. The reaction equation is shown in Figure 1 and the specific synthesis process is as follows: ① Weigh 100 g of PTMG and place it in a three-necked flask, then put the flask in an oven and vacuum dry PTMEG at 120 °C for 2 h; ② After drying, place the flask in an 80 °C oil bath and add 18.24 g IPDI to it; ③ Add 2 drops of catalyst ditin butyl dilaurate (DBTDL) to the system and react for 60 min to obtain a pre-polymer; ④ Dissolve 6.76 g of DPA in 15 mL of dimethylformamide (DMF) solvent and add it to the reaction system for 30 min; ⑤ After the reaction is completed, pour PU into a polytetrafluoroethylene mold, then dry it in an oven at 80 °C for 48 h to completely evaporate the solvent DMF.

Synthesis for PU/GO: the synthesis process of PU/GO is similar to PU, but GO needs to be introduced into the pre-polymer before chain extension. The specific synthesis process is as follows: ① Weigh 100 g of PTMG and place it in a three-necked flask, then put the flask in an oven and vacuum dry PTMEG at 120 °C for 2 h; Weigh the corresponding mass of GO (0.4%, 0.8%, 1.2%, 1.6%) and add it to DMF solvent, then use ultrasonic to evenly disperse GO for 2 h; ② After drying, place the flask in an 80 °C oil bath and add the weighed IPDI (18.24 g) to it; ③ Add 2 drops of DBTDL to the system and react for 60 min to obtain a pre-polymer; ④ Add GO dispersion to the pre-polymer and allow the hydroxyl and carboxyl groups on GO to react with isocyanate groups for 30 min; ⑤ Dissolve 6.76 g of DPA in 15 mL of DMF solvent and add it to the reaction system for 30 min; ⑥ After the reaction is completed, pour PU/GO into a polytetrafluoroethylene mold, then dry it in an oven at 80 °C for 48 h to completely evaporate the solvent DMF.

#### 2.2.2. The Preparation of Modified Asphalt

**Figure 2:**
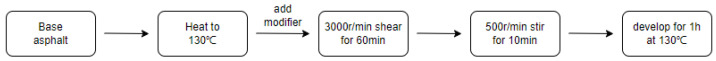
The specific process for preparing modified asphalt.

In order to blend the modifier with the asphalt uniformly and fully utilize the function of the modifier, FM300 high-speed shear apparatus was chosen to prepare modified asphalt in this study. According to a preliminary experiment, shearing at a rate of 3000 r/min for 60 min can evenly disperse the modifier in asphalt. The specific preparation process is shown in Figure 2.

### 2.3. Test Methods

#### 2.3.1. Fourier Transform Infrared (FTIR) Test

The FTIR test is commonly used to analyze the functional groups and molecular structures of chemical compounds. Due to the unique vibration frequencies of different chemical bonds, the FTIR test can be used to determine the chemical bonds in molecules. Therefore, the Thermo Fisher Scientific Nicolet iN10 FTIR spectrometer from the Waltham, MA, USA was used to determine the functional groups and molecular structure composition of PU and PU/GO, and the scanning wavelengths ranged from 400 ${\mathrm{cm}}^{-1}$ to 4000 ${\mathrm{cm}}^{-1}$.

#### 2.3.2. Field Emission Scanning Electron Microscope (SEM) Test

The micro morphological structure information of various solid-state sample surfaces can be obtained through SEM tests. Therefore, in order to investigate the effect of GO contents on the apparent morphology of PU and the dispersion conditions of GO in PU, a SEM test was selected to observe the microstructure of PU/GO. In this study, the sample will be observed at a magnification of 400 times.

#### 2.3.3. Gel Permeation Chromatography (GPC) Test

GPC test is based on the basic principles of solvent dissolution, adsorption, diffusion, etc. Through the GPC test, the molecular weight size and distribution of different molecules can be determined, and parameters such as number average molecular weight ($M_{n}$), weight average molecular weight ($M_{W}$), and polydispersity (PD) coefficient can be obtained. In this study, the molecular weight of PU with different GO contents was tested through GPC.

#### 2.3.4. Mechanical and Self-Healing Test of PU and PU/GO

**Figure 3:**
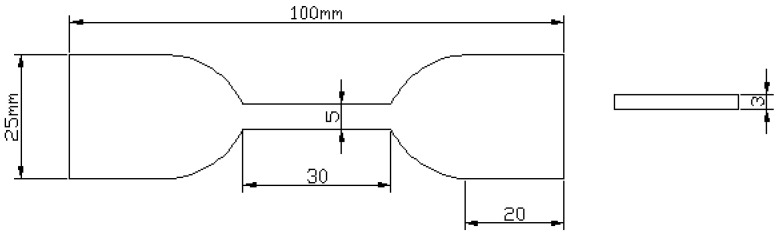
Dimensions of the specimen for tensile test.

In order to characterize the mechanical properties of PU and PU/GO, a tensile test was conducted. PU/GO material was prepared as dumbbell-shaped standard specimens for tensile testing according to the standard GB/T 528-2009 [38], and the dimensions of the specimen are shown in Figure 3. Before the start of the test, 0.1N pre-load was applied to the specimen to ensure that the initial state of the specimen was consistent and the loading rate was set as 500 mm/min.

The mechanical properties of PU/GO materials are mainly evaluated based on two indicators: tensile strength σ and breaking elongation e. The calculation formula is as follows:

(1) $$\sigma =\frac{F_{b}}{S_{0}}$$

(2) $$e=\frac{\left(L-L_{0}\right)}{L_{0}}\times 100\%$$

where $F_{b}$ is the maximum tensile axial force, and $S_{0}$ is the initial cross-sectional area of the specimen, L is the length of the section when the specimen is broken, and $L_{0}$ is the original length of the section.

**Figure 4:**
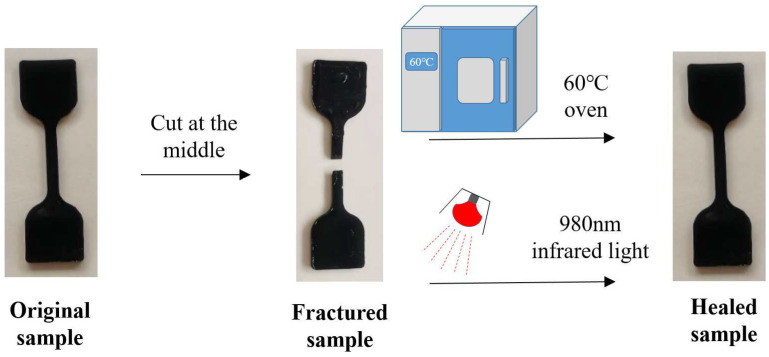
Self-healing test of PU/GO.

The self-healing performance of PU/GO is obtained by comparing the tensile strength of the specimens before and after self-healing. The PU/GO specimen will be cut in the middle by a knife, then let the fracture surface contact with each other. The fracture specimens will be placed in an environment of 60 °C for different times (1 h, 2 h, 4 h) or exposed to infrared light with a wavelength of 980 nm with different times (5 min, 10 min, 15 min) for self-healing. The specific process of the test is shown in Figure 4 and the self-healing coefficient $H_{\sigma }$ can be calculated as follows:

(3) $$H_{\sigma }=\frac{\sigma_{1}}{\sigma_{0}}\times 100\%$$

where $\;\sigma_{1}$ is the tensile strength of the specimens after fracture and self-healing, $\sigma_{0}$ is the tensile strength of the original specimen.

#### 2.3.5. Fluorescence Microscopy (FM) Test

FM test uses ultraviolet light as a light source to make the sample display fluorescence, and then the shape and position of the sample can be observed under the microscope. Asphalt and polymer display different brightnesses, so the dispersion of polymer in asphalt can be observed.

#### 2.3.6. Basic Performance of Modified Asphalt

The conventional indices such as penetration, softening point, ductility and viscosity of modified asphalt were tested according to the specification JTG E20-2011 [37].

#### 2.3.7. Self-Healing Test of Modified Asphalt

**Figure 5:**
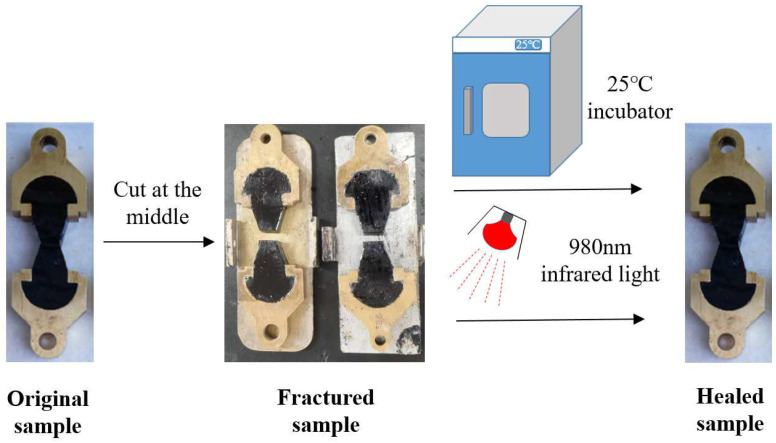
Self-healing test of asphalt based on ductility test.

The self-healing performance of modified asphalt was evaluated by two methods: ductility test and bending beam rheometer (BBR) test. The specimens of ductility test and BBR test will be cut in the middle, then the fractured surfaces will be allowed to be in contact with each other. The fractured specimens will be placed in an environment of 25 °C for different times (1 h, 2 h, 4 h) for self-healing or exposed to infrared light with a wavelength of 980 nm for different times (5 min, 10 min, 15 min) for self-healing. The specific process of the asphalt self-healing test based on the ductility test is shown in Figure 5. The ductility test was conducted at 5 °C and the self-healing coefficient $H_{L}$ can be calculated as follows:

(4) $$\;H_{L}=\frac{L_{1}}{L_{0}}\times 100\%$$

where $\;L_{1}$ is the ductility of the specimen after fracture and self-healing, $L_{0}$ is the ductility of the original specimen.

**Figure 6:**
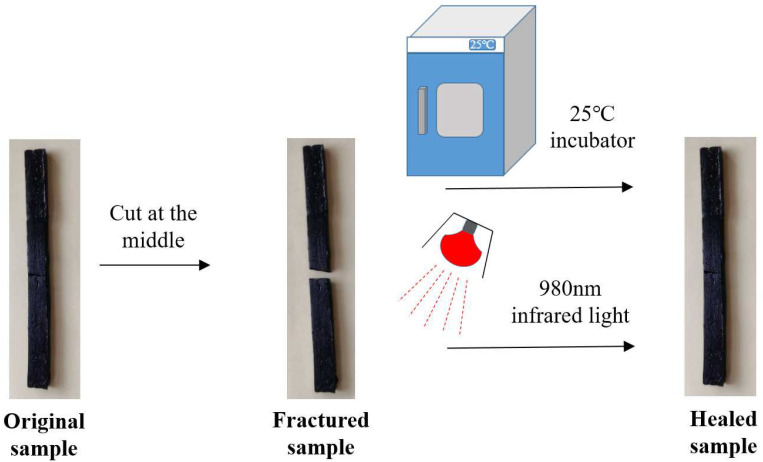
Self-healing test of asphalt based on BBR test.

The specific process of the asphalt self-healing test based on the BBR test is shown in Figure 6. The BBR test was conducted at −12 °C and the self-healing coefficient $H_{S}$ can be calculated as follows:

(5) $$\;H_{S}=\frac{S_{1}}{S_{0}}\times 100\%$$

where $\;S_{1}$ is the stiffness modulus of the specimen after fracture and self-healing, $S_{0}$ is the stiffness modulus of the original specimen.

### 2.4. Study Procedures

**Figure 7:**
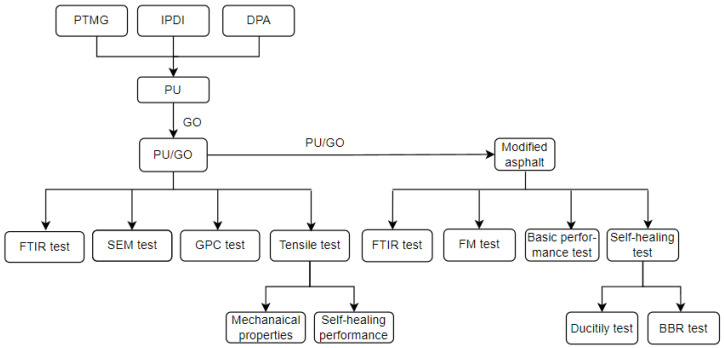
The complete process of this study.

The specific study procedures are shown in Figure 7.

## 3. Results and Discussion

### 3.1. Performance Characterization of PU/GO

#### 3.1.1. FTIR Test of PU/GO

**Figure 8:**
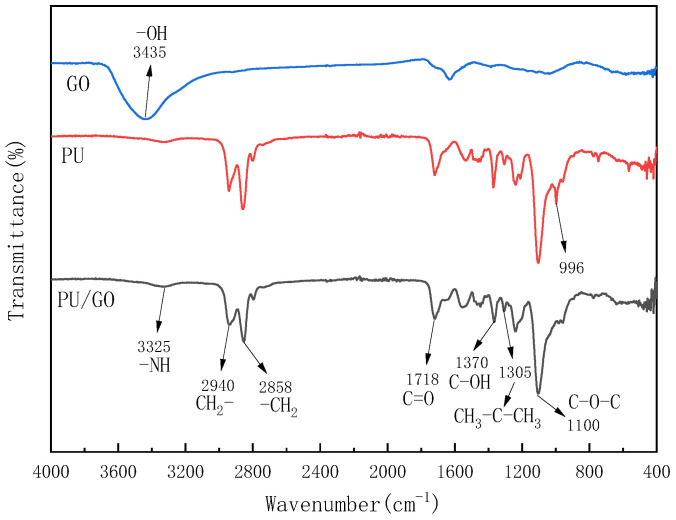
The FTIR spectra of PU and PU/GO.

From the FTIR spectra of PU/GO in Figure 8, it can be found that an absorption peak appears at 3325 ${\mathrm{cm}}^{-1}$ due to the stretching vibration of –NH. And the hydroxyl group (−OH) can be found in GO spectra at 3435 ${\mathrm{cm}}^{-1}$ but cannot be found around 3400 ${\mathrm{cm}}^{-1}$ in the spectra of PU/GO, which indicates that the –OH reacted to generate –NH. The absorption peaks of 2940 ${\mathrm{cm}}^{-1}$ and 2858 ${\mathrm{cm}}^{-1}$ belong to $-{\mathrm{CH}}_{2}$. The stretching vibration frequency of carboxylic acid carbonyl C=O is between 1760–1660 ${\mathrm{cm}}^{-1}$, while the vibration frequency of aliphatic carboxylic acid carbonyl is around 1700 ${\mathrm{cm}}^{-1}$. Therefore, the peak at 1718 ${\mathrm{cm}}^{-1}$ is the stretching vibration peak of carboxylic acid carbonyl in DPA. In addition, C−OH in the carboxyl group will also produce stretching vibration, corresponding to the vibration peak at 1305 ${\mathrm{cm}}^{-1}$. When two or three $-{\mathrm{CH}}_{3}$ groups are connected to the same carbon atom, a coupling effect will occur in the symmetric angular vibration of $-{\mathrm{CH}}_{3}$. The IPDI has a structure where two methyl groups are connected to the same carbon atom. Therefore, the absorption peak of 1305 ${\mathrm{cm}}^{-1}$ belongs to the angular vibration of $-{\mathrm{CH}}_{3}$ in IPDI. In addition, the absorption peak 1100 ${\mathrm{cm}}^{-1}$ belongs to C−O−C. What is more, the absorption peak 2250$\;{\mathrm{cm}}^{-1}$ and 1350 ${\mathrm{cm}}^{-1}$ belong to −NCO can not be found in the spectra, which proves that the −NCO functional groups have reacted.

#### 3.1.2. SEM Test of PU/GO

**Figure 9:**
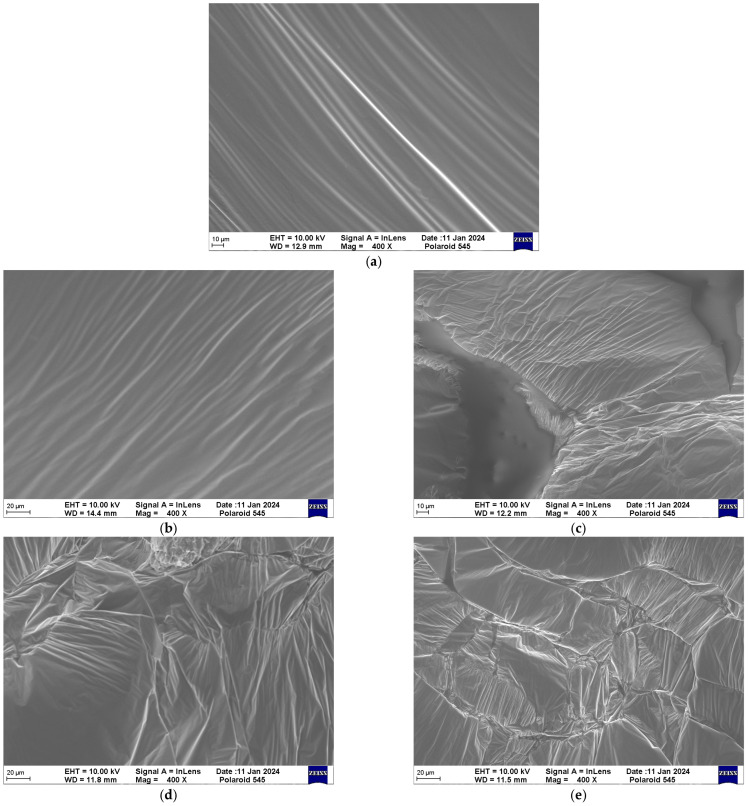
The micromorphology of PU with different GO contents. (**a**) 0% GO content. (**b**) 0.4% GO content. (**c**) 0.8% GO content. (**d**) 1.2% GO content. (**e**) 1.6% GO content.

Figure 9 shows the micromorphology of PU with different GO contents. It can be seen that in Figure 9a, the microstructure of pure PU is very regular, presenting parallel strip-shaped ripples. It can be inferred that the arrangement of molecular chains in PU is also parallel, with less cross-linking between molecular chains. When the GO content is 0.4%, the microstructure is similar to that of pure PU, showing regular stripe shapes. When the GO content increases to 0.8%, most of the molecular chains still exhibit a striped arrangement. But some molecular chains crosslink with each other and result in a certain degree of irregularity in the microstructure. When the GO content continues to increase to 1.2% or even 1.6%, the spatial cross-linking between molecular chains further develops, resulting in multiple discontinuous interfaces. It can be concluded that the irregularity of the PU/GO microstructure will increase with the increase in GO content. The reason can be attributed to the spatial structure of GO. Compared with PTMEG, GO has a typical two-dimensional spatial structure rather than a linear structure, which will have an effect on the linear spatial structure of molecular chains.

#### 3.1.3. GPC Test of PU/GO

Table 6 shows the GPC test results of PU/GO with different GO contents. It can be seen that the $M_{W}$ of PU/GO with different GO contents range from 30,000–60,000, which indicates that the molecules of raw material react with each other successfully and generate macromolecular polymers. The PD of PU/GO with different GO contents ranges from 1 to 3, which shows that the molecular weight distribution is relatively uniform. The results also show that the molecular of PU/GO decreases with the increase in GO content and the PD is also positively correlated with GO content. It may be related to the molecular structure of GO. GO has a typical two-dimensional spatial structure rather than a linear structure, which will have an impact on the linear structure of the molecular chain. Moreover, there are many hydroxyl and carboxyl groups on the surface of GO, which can react with diisocyanate groups. The two isocyanate groups in the same molecular chain may react with functional groups of the same GO molecule, which will affect the extension of the molecular chain.

#### 3.1.4. Mechanical Properties of PU/GO

**Figure 10:**
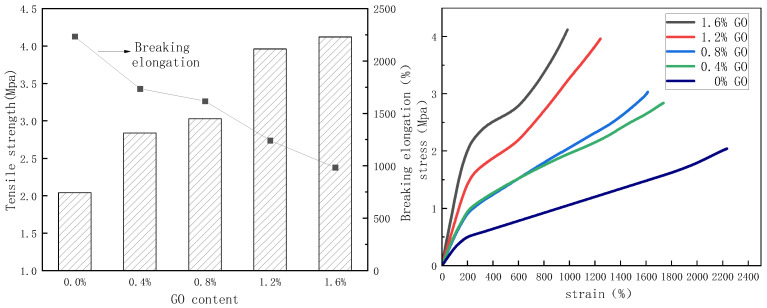
The mechanical property results of PU/GO.

According to Figure 10, it can be found that the effect of different GO content on the mechanical properties of PU/GO. The tensile strength of PU/GO increases with the increase in GO content while the breaking elongation gradually decreases. Compared with pure PU, the stress of PU/GO with 1.6% GO content increases by 102%, which indicates that the introduction of GO has a significant enhancing effect on the strength of PU. The reason can be attributed to the excellent mechanical properties of GO. When the GO is introduced into PU prepared reaction system, the hydroxyl and carboxyl groups on GO will react with isocyanate groups and GO will be embedded in the molecular chain of PU. But at the same time, the addition of GO will reduce the breaking elongation of PU, because the introduction of GO will affect the linear structure of the molecular chain to some extent.

#### 3.1.5. Self-Healing Properties of PU/GO

**Figure 11:**
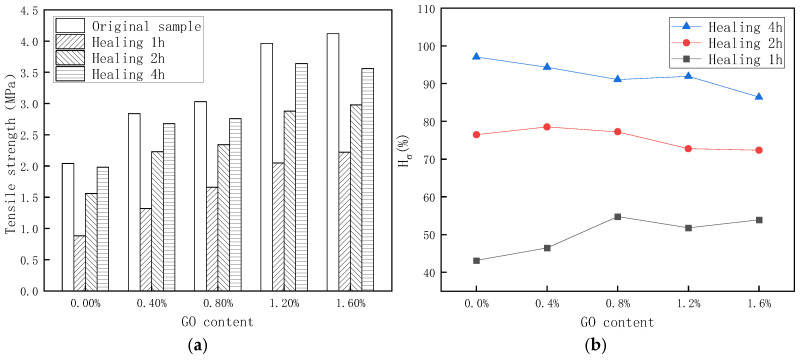
The self-healing test results of PU/GO under 60 °C condition. (**a**) Stress of healing sample (**b**) Self-healing coefficient.

**Figure 12:**
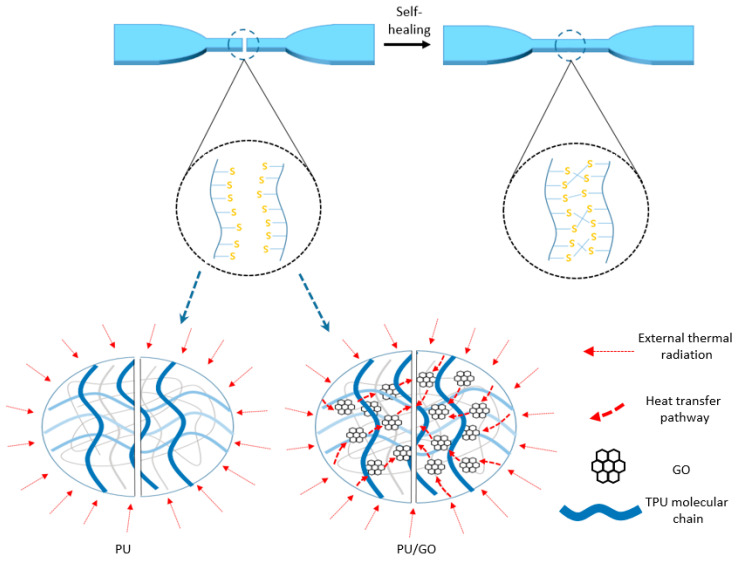
Self-healing mechanism of PU and PU/GO.

From Figure 11, the impact of healing time and different GO contents on the self-healing property of PU/GO can be understood. Moreover, the self-healing mechanism and process are shown in Figure 12. When the fractured surfaces of the specimen come into contact with each other, the molecular chains around the fractured surfaces will move and the disulfide bonds around the surfaces will recombine to heal the fracture. The movement of molecular chains and the recombination of disulfide bonds will continue to increase over time. Therefore, it can be seen from Figure 11a that the strength of the healing sample gradually increases with the increase in healing time. What is more, molecular chains will also intertwine with each other with the increase in healing time, which is beneficial to improve the strength of the sample. On the other hand, the movement of molecular chains and the recombination speed of disulfide bonds are both affected by temperature; the higher the temperature, the higher the self-healing efficiency. It can be seen in Figure 12 that, compared with pure PU, lamellar structure GO is dispersed in PU/GO, and this can improve the thermal conductivity of the sample. Compared with pure PU, the temperature of the fractured surfaces of the PU/GO sample is higher, which is beneficial for improving the recombination efficiency of disulfide bonds. But at the same time, the movement of molecular chains will be restricted to some extent due to the lamellar structure of GO, which will have adverse effects on the self-healing of the sample. These two factors will jointly affect the self-healing properties of PU/GO. So, it can be found that from Figure 11b, the self-healing coefficient of sample healing for 1 h increases with the increase in GO content while the self-healing coefficient of sample healing for 2 h and 4 h decreases. And the self-healing coefficient of PU/GO sample healing for 1 h does not keep increasing with the increase in GO content. When the GO content is 0.8%, the self-healing coefficient reaches the maximum. The results demonstrate that the addition of GO has a positive impact on the early self-healing property of the sample but a negative impact on the final self-healing results.

**Figure 13:**
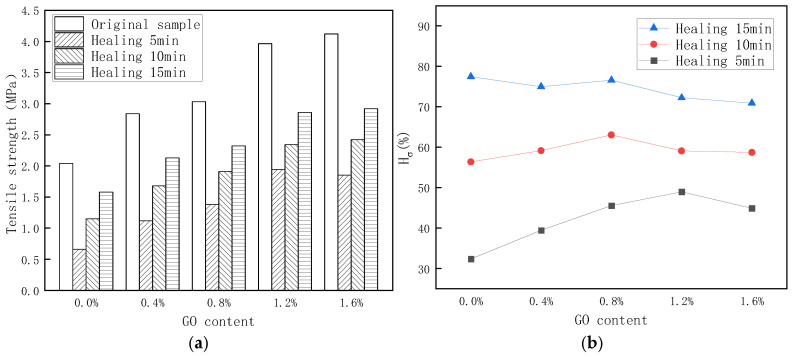
The self-healing test results of PU/GO under infrared light conditions. (**a**) Stress of healing sample (**b**) Self-healing coefficient.

Figure 13 shows that the results under infrared light conditions are similar to that under 60 °C conditions. With the increase in healing time, the tensile strength of PU/GO samples with different GO content gradually increases. When the healing time is 5 min, it can be discovered that when the GO content increases, the self-healing coefficient gradually increases until the GO content reaches 1.2%. The results also show that under infrared light conditions, the addition of GO improves the early-stage self-healing property of the sample but decreases the final self-healing results. In the early stage, the sample with higher GO content will have a higher temperature because of the excellent infrared light absorption ability of GO, which is beneficial for self-healing performance. However, the adverse effects of GO on restricting the movement of molecular chains will gradually manifest.

### 3.2. Performance Characterization of PU/GO-Modified Asphalt

#### 3.2.1. FTIR Test of PU/GO-Modified Asphalt

**Figure 14:**
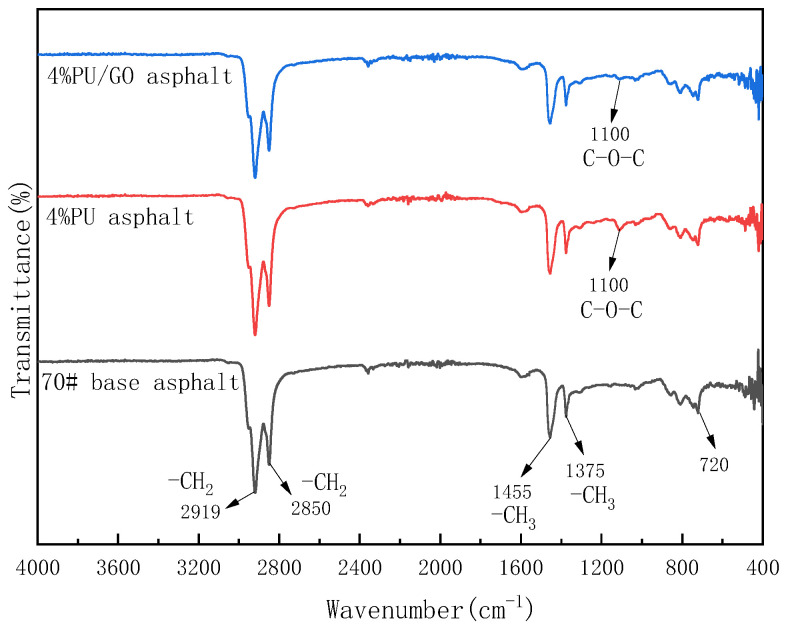
The FTIR test results of base asphalt and modified asphalt.

According to Figure 14, the absorption peaks of 2919 ${\mathrm{cm}}^{-1}$ are the symmetric stretching vibration of C−H in $-{\mathrm{CH}}_{2}$ and 2850 ${\mathrm{cm}}^{-1}$ is the asymmetric stretching vibration of C−H in $-{\mathrm{CH}}_{2}$. The vibration peak 1455 ${\mathrm{cm}}^{-1}$ is the in-plane stretching vibration of C−H in $-{\mathrm{CH}}_{3}$ and 1375 ${\mathrm{cm}}^{-1}$ is the in-plane stretching vibration of C−H in $-{\mathrm{CH}}_{3}$. The absorption peaks of 720 ${\mathrm{cm}}^{-1}$ belong to the out-of-plane bending vibration of C-H on the benzene ring. When it comes to the spectra of PU-modified asphalt, it can be found that the main vibration peaks in the spectra are similar to base asphalt. But different from base asphalt, there is a new vibration peak at 1100 ${\mathrm{cm}}^{-1}$ in the spectra of PU-modified asphalt. It can be discovered that the vibration peak at 1100 ${\mathrm{cm}}^{-1}$ belongs to PU according to the spectra of PU materials. What is more, the spectra of PU/GO-modified asphalt are almost the same as that of PU-modified asphalt, which indicates that both PU and PU/GO have no reaction with asphalt.

#### 3.2.2. FM Test Results of PU/GO-Modified Asphalt

**Figure 15:**
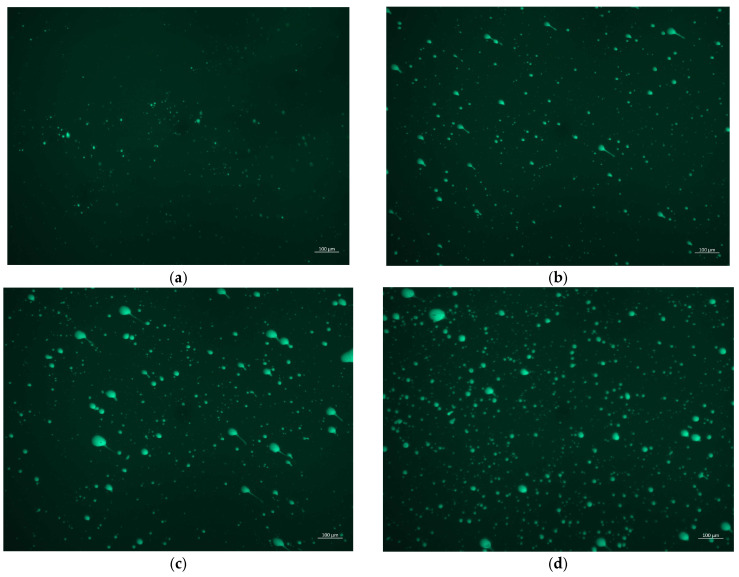
The FM image of PU/GO-modified asphalt. (**a**) 2% PU/GO content (**b**) 4% PU/GO content. (**c**) 6% PU/GO content (**d**) 8% PU/GO content.

According to the principle of FM test, it can be understood that the black area is asphalt, and the fluorescence is the PU/GO modifier. From Figure 15, the dispersion of PU/GO polymer in asphalt can be observed. When the PU/GO content is 2%, the overall brightness is relatively low, and the modifier particles are relatively small. The result reveals that the PU/GO can be evenly dispersed in asphalt. When the content of PU/GO increases to 4%, the overall brightness significantly increases, with more fluorescent dots appearing in the figure. The modifier PU/GO can still be evenly dispersed in asphalt. As the PU/GO content continues to increase, the brightness of the fluorescence image increases, and the bright spots become denser. When the PU/GO content is 8%, although some larger particles can be seen on the fluorescence image, it can also be observed that the vast majority of modifiers are uniformly dispersed in the asphalt. Based on the above discussion, it can be concluded that with the increase in modifier PU/GO dosage, the brightness of the fluorescence image gradually increases. And the PU/GO can be uniformly dispersed in the asphalt, which indicates that PU/GO has good compatibility with asphalt. The reason may be related to the physical properties of PU/GO. The density of PU/GO is similar to that of asphalt and its melting point is lower than modified asphalt preparation temperature, so PU/GO could be well compatible with asphalt.

#### 3.2.3. Basic Properties of Modified Asphalt

Table 7 shows the basic properties of PU-modified asphalt (PA) and PU/GO-modified asphalt (PGA). The penetration of PA gradually increases with the increase in PU content while the penetration of PU/GO-modified asphalt gradually decreases, which indicates PU has a softening effect on asphalt at room temperature, while PU/GO makes asphalt harder at room temperature. This is because the addition of GO in PU will enhance the mechanical properties of PU and make PU harder at room temperature. On the other hand, the softening points of PA and PGA are only slightly improved compared with base asphalt. But it can be observed that the softening point of PGA is higher than PA, which shows that the high-temperature performance of PGA is greater than PA. What is more, both PU and PU/GO can significantly improve the low-temperature flexibility of asphalt and the effect of PU is more obvious than PU/GO. The reason can be attributed to the excellent flexibility of PU. Both the high-temperature viscosities of PU and PU/GO-modified asphalt gradually increase with the increase in modifier content, but the improvement is not significant. Through the test results in Table 6, it can be concluded that PU and PU/GO have an obvious improvement influence on low-temperature performance, while the impact on high-temperature performance is not significant.

#### 3.2.4. Self-Healing Test Results of Modified Asphalt

##### Self-Healing Performance Base on Ductility Test

**Figure 16:**
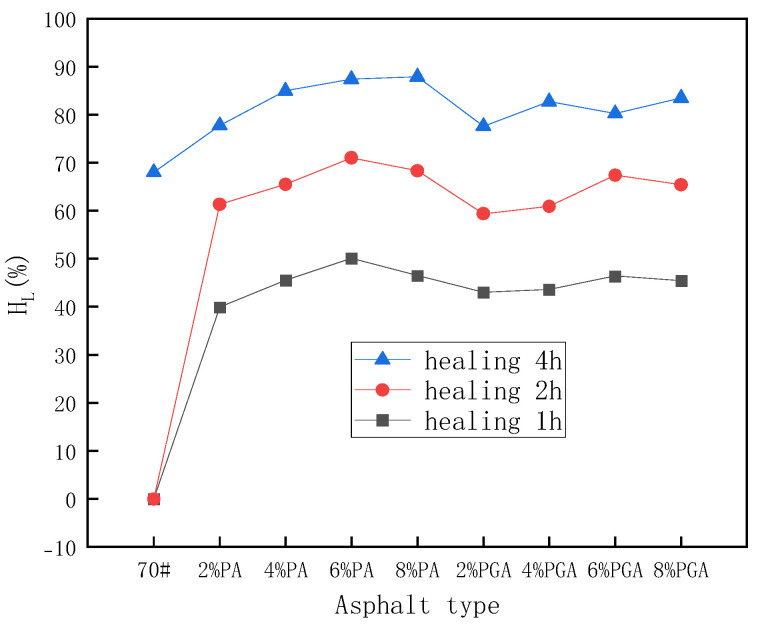
The ductility self-healing coefficient results under 25 °C conditions.

Table 8 displays the ductility results before and after self-healing under 25 °C conditions. It can be found that after self-healing for 1 h or 2 h, the ductility of base asphalt cannot be measured. When the healing time increases to 4 h, the ductility is restored to 5.1 cm, which indicates that base asphalt has self-healing ability to a certain extent. Moreover, the ductility of all the samples increases gradually as the healing time increases, which proves that healing time plays an important role in the self-healing process. According to Figure 16, it can be discovered that both PU and PU/GO have a positive impact on the self-healing performance of asphalt. The overall trend of the self-healing coefficient increases with the increase in the PU and PU/GO content. This is because with the increase in PU and PU/GO content, the quantity of disulfide covalent bonds in asphalt will also increase. Therefore, the recombination efficiency of disulfide bonds will increase and have a positive impact on the self-healing property. What is more, the self-healing coefficients of PU-modified asphalt are higher than PU/GO-modified asphalt, which indicates that under room temperature, PU has a more significant influence on self-healing properties.

**Figure 17:**
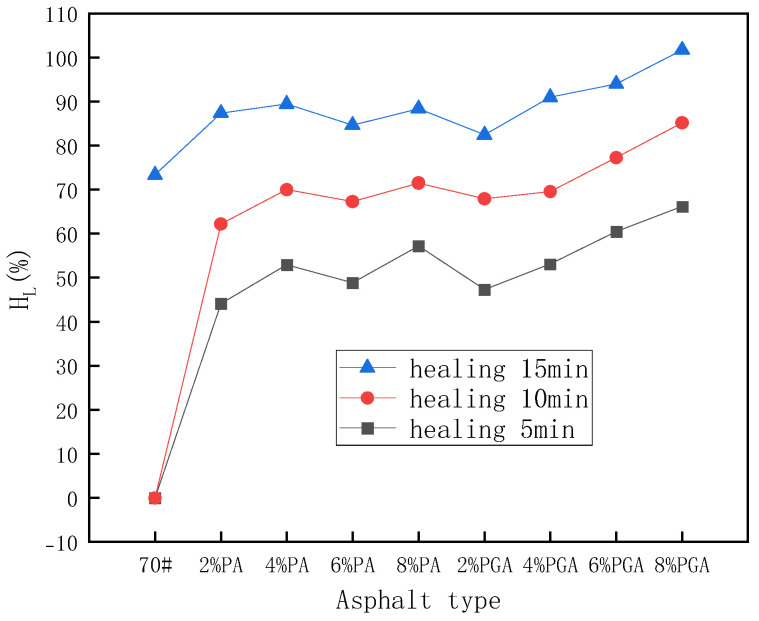
The ductility self-healing coefficient results under infrared light conditions.

Table 9 and Figure 17 shows the ductility results before and after self-healing under infrared light conditions. It can be also seen that the addition of PU and PU/GO can improve the self-healing coefficient. But the results are not totally the same as the results under 25 °C conditions. Under infrared light conditions, the self-healing coefficients of PU/GO-modified asphalt are higher than that of PU-modified asphalt when the modifier content is 6% or 8%. When the healing time is 15 min under infrared light conditions, the self-healing coefficient of 8%PU/GO-modified asphalt is greater than 100%, which indicates that the fracture was completely repaired. The reason can be attributed to the excellent infrared light absorption ability. A higher infrared light absorption rate will increase the temperature of asphalt, resulting in better self-healing ability.

##### Self-Healing Performance Base on BBR Test

According to Table 10, it can be found that with the increase in healing time, the stiffness modulus of all the fractured-healing samples gradually increases, which proves that healing time plays an important role in the asphalt self-healing process.

**Figure 18:**
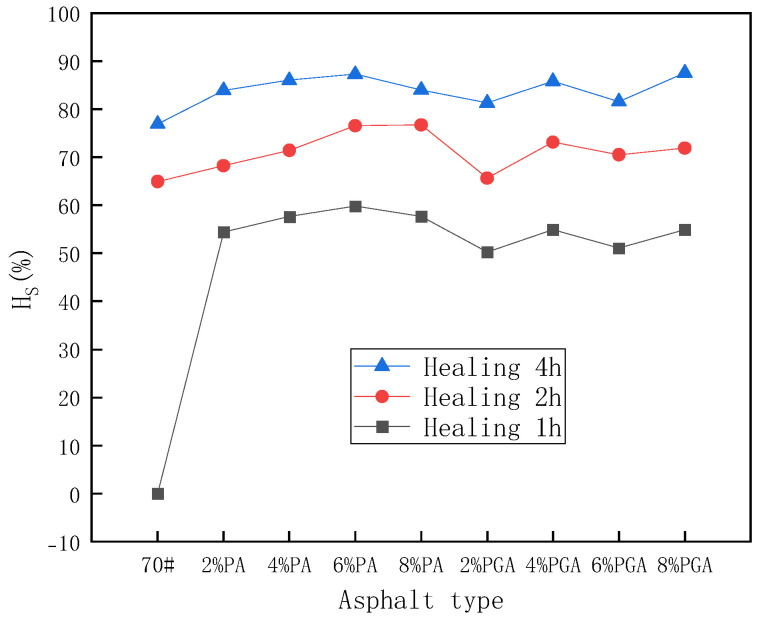
The stiffness modulus self-healing coefficient results under 25 °C conditions.

According to Figure 18, it can be seen that, similar to the results of the ductility test, the${\;H}_{S}$ of PU and PU/GO-modified asphalt is higher than base asphalt. What is more, the higher the modifier content, the higher the self-healing coefficient is, which shows that both PU and PU/GO have a positive effect on the self-healing properties. When it comes to the results of the BBR test, it can also be concluded that the effect of PU on self-healing properties is more significant than PU/GO at room temperature. The conclusion is consistent with that of the ductility test.

**Figure 19:**
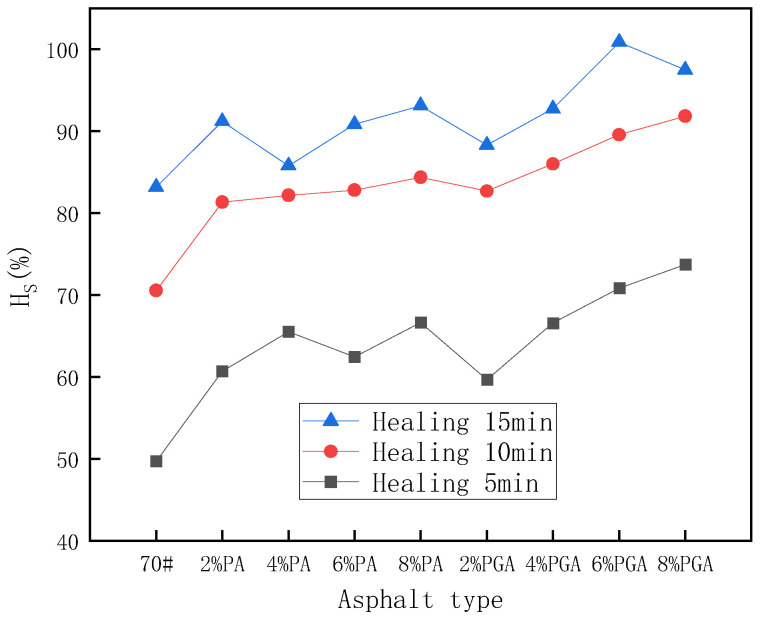
The stiffness modulus self-healing coefficient results under infrared light conditions.

Table 11 and Figure 19 show the self-healing test results under infrared light conditions based on the BBR test. Similar to the ductility test, the results also show that the addition of PU and PU/GO can increase the self-healing properties under infrared light conditions. But this is different from room temperature conditions; the impact of PU/GO on self-healing properties is more obvious than PU under infrared light conditions. Compared with 6% PA, the self-healing coefficient of 6% PGA reaches 100.8% when the healing time is 15 min, which is 10.0% higher than that of 6% PA. At room temperature conditions, the PU-modified asphalt has better mobility than PU/GO-modified asphalt, which will result in better self-healing properties. But under infrared light conditions, the temperature of PU/GO-modified asphalt will be higher than PU-modified asphalt due to the better infrared light absorption ability of PU/GO. Higher temperature is beneficial for the self-healing of asphalt.

## 4. Conclusions

In this study, a composite material, PU/GO, containing dynamic disulfide bonds, was synthesized and used to modify asphalt. The basic and self-healing properties of PU/GO and PU/GO-modified asphalt were explored through FTIR, SEM, GPC, FM and self-healing tests. The key conclusions are as follows:

1. FTIR test results showed that PU/GO was successfully synthesized and the introduction of GO in PU increased the irregularity of PU micromorphology and decreased the molecular weight of PU.
2. GO could significantly improve the mechanical properties of PU. Due to its excellent thermal conductivity and infrared absorption capability, GO had a positive impact on the early self-healing property of PU under 60 °C conditions and infrared light conditions.
3. GO had a slight negative impact on the final self-healing results because it hindered the movement of molecular chains to a certain extent.
4. PU/GO could be well dispersed in asphalt and significantly improve the low-temperature flexibility of base asphalt.
5. Both ductility and BBR tests showed that PU and PU/GO improved the self-healing properties of asphalt due to the introduction of a disulfide bond. PU had a more obvious influence on the self-healing properties at room temperature because of its excellent flexibility, while PU/GO had a more significant impact under infrared light conditions due to its better thermal conductivity.

This study mainly evaluates the self-healing performance of PU/GO and PU/GO-modified asphalt through macroscopic experiments, which is lacking exploration at the microscopic and molecular levels. In the following research, it is necessary to explore the self-healing mechanism of GO/PU and asphalt at the molecular level.

## Figures and Tables

**Table 1:** Relevant indices of PTMEG.

Index	Unit	Result	Index	Unit	Result
Molecular weight	/	2000	Melting point	°C	33
Density	g/cm^3^	0.972	Hydroxyl value	$\mathrm{mgKOH}\cdot g^{-1}$	56
Viscosity (40 °C)	mPa·s	1120	Water content	%	≤0.05
CAS Registry Number		25190-06-1			

**Table 2:** Relevant indices of IPDI.

Index	Unit	Result	Index	Unit	Result
Molecular weight	/	222	Melting point	°C	−60
Density	g/cm^3^	1.062	Boiling point	°C	158
Viscosity (25 °C)	mPa·s	15	NCO content	%	≥37.5
CAS Registry Number		4098-71-9			

**Table 3:** Relevant indices of DPA.

Index	Unit	Result	Index	Unit	Result
Molecular weight	/	210	Melting point	°C	156
Density	g/cm^3^	1.452	Boiling point	°C	431
CAS Registry Number		1119-62-6			

**Table 4:** Basic properties of 70 pen base binder.

Index	Unit	Test Results	Requirement	Test Method
Ductility (10 °C, 5 cm/min)	cm	44.2	>25	T 0605-2011
Ductility (15 °C, 5 cm/min)	cm	>150	>100	T 0605-2011
Penetration (25 °C, 100 g, 5 s)	0.1 mm	65.5	60–80	T 0604-2011
$\mathrm{Softening}\;\mathrm{point}\;\left(R\&B\right)$	°C	46.4	>46	T 0606-2011
Viscosity (135 °C)	Pa·s	0.49	−	T 0625-2011
Elastic recovery (25 °C)	%	16	−	T 0662-2011

**Table 5:** Recipes for the preparation of PU and PU/GO.

	Hard Segment Content	PTMEG (g)	IPDI (g)	DPA (g)	GO (g)
PU	20%	100 (0.05 mol)	18.24 (0.082 mol)	6.76 (0.032 mol)	−
PU/GO	20%	100 (0.05 mol)	18.24 (0.082 mol)	6.76 (0.032 mol)	0.5, 1.0, 1.5, 2.0

**Table 6:** The GPC test results of PU/GO.

GO Content	$\mathrm{Number}\;\mathrm{Average}\;\mathrm{Molecular}\;\mathrm{Weight}/M_{n}$	$\mathrm{Weight}\;\mathrm{Average}\;\mathrm{Molecular}\;\mathrm{Weight}/M_{W}$	Polydispersity/PD
0	40,230	56,121	1.395
0.4%	26,054	48,252	1.852
0.8%	24,820	41,326	1.665
1.2%	15,434	36,285	2.351
1.6%	16,857	38,165	2.264

**Table 7:** The basic properties test results of modified asphalt.

Asphalt Type	Penetration (25 °C)/0.1 mm	Softening Point/°C	Ductility (5 °C)/cm	Viscosity (135 °C)/Pa·s
70#	65.5	46.4	7.5	0.49
2%PA	64.6	46.8	23.8	0.50
4%PA	70.2	46.0	40.6	0.52
6%PA	71.5	47.2	48.3	0.54
8%PA	72.6	47.0	56.8	0.57
2%PGA	63.5	46.6	16.5	0.53
4%PGA	61.2	47.0	24.3	0.55
6%PGA	60.5	48.2	33.4	0.62
8%PGA	58.8	48.6	40.5	0.68

**Table 8:** Ductility self-healing test results under 25 °C condition.

Asphalt Type	Original Sample/cm	Healing 1 h/cm	Healing 2 h/cm	Healing 4 h/cm
70#	7.5	−	−	5.1
2%PA	23.8	9.5	14.6	18.5
4%PA	40.6	18.5	26.6	34.5
6%PA	48.3	24.2	34.3	42.2
8%PA	56.8	26.4	38.8	49.9
2%PGA	16.5	7.1	9.8	12.8
4%PGA	24.3	10.6	14.8	20.1
6%PGA	33.4	15.5	22.5	26.8
8%PGA	40.5	18.4	26.5	33.8

**Table 9:** Ductility self-healing test results under infrared light conditions.

Asphalt Type	Original Sample/cm	Healing 5 min/cm	Healing 10 min/cm	Healing 15 min/cm
70#	7.5	−	−	5.5
2%PA	23.8	10.5	14.8	20.8
4%PA	40.6	21.5	28.4	36.3
6%PA	48.3	23.6	32.5	40.9
8%PA	56.8	32.5	40.6	50.2
2%PGA	16.5	7.8	11.2	13.6
4%PGA	24.3	12.9	16.9	22.1
6%PGA	33.4	20.2	25.8	31.4
8%PGA	40.5	26.8	34.5	41.2

**Table 10:** Stiffness modulus self-healing test results under 25 °C condition.

Asphalt Type	Original Sample/MPa	Healing 1 h/MPa	Healing 2 h /MPa	Healing 4 h /MPa
70#	111.374	−	72.325	85.652
2%PA	92.535	50.365	63.153	77.654
4%PA	84.364	48.636	60.251	72.586
6%PA	80.656	48.253	61.753	70.385
8%PA	81.256	46.856	62.352	68.253
2%PGA	101.325	50.963	66.522	82.364
4%PGA	93.325	51.263	68.258	80.063
6%PGA	94.524	48.259	66.653	77.132
8%PGA	85.321	46.896	61.356	74.698

**Table 11:** Stiffness modulus self-healing test results under infrared light conditions.

Asphalt Type	Original Sample/MPa	Healing 5 min/MPa	Healing 10 min/MPa	Healing 15 min/MPa
70#	111.374	55.362	78.569	92.623
2%PA	92.535	56.149	75.258	84.378
4%PA	84.364	55.268	69.315	72.354
6%PA	80.656	50.365	66.765	73.254
8%PA	81.256	54.149	68.554	75.656
2%PGA	101.325	60.449	83.761	89.443
4%PGA	93.325	62.118	80.249	86.544
6%PGA	94.524	66.964	84.653	95.321
8%PGA	85.321	62.883	78.358	83.149

## Data Availability

The original contributions presented in this study are included in the article. Further inquiries can be directed to the corresponding author.
